# Pharmacological elevation of sphingosine‐1‐phosphate by S1P lyase inhibition accelerates bone regeneration after post‐traumatic osteomyelitis

**DOI:** 10.1111/jcmm.17952

**Published:** 2023-09-14

**Authors:** Johannes M. Wagner, Annalena Wille, Maria Fueth, Sarah Weske, Sebastian Lotzien, Felix Reinkemeier, Christoph Wallner, Alexander Sogorski, Stephanie Dittfeld, Mustafa Becerikli, Thomas A. Schildhauer, Marcus Lehnhardt, Bodo Levkau, Björn Behr

**Affiliations:** ^1^ Department of Plastic Surgery BG University Hospital Bergmannsheil Bochum Bochum Germany; ^2^ Department of Trauma Surgery and General Surgery BG University Hospital Bergmannsheil Bochum Bochum Germany; ^3^ Institute of Molecular Medicine III University Hospital Düsseldorf and Heinrich Heine Universität Düsseldorf Düsseldorf Germany

**Keywords:** bone regeneration, osteomyelitis, sphingosin‐1‐phosphate

## Abstract

Posttraumatic osteomyelitis and the ensuing bone defects are a debilitating complication after open fractures with little therapeutic options. We have recently identified potent osteoanabolic effects of sphingosine‐1‐phosphate (S1P) signalling and have now tested whether it may beneficially affect bone regeneration after infection. We employed pharmacological S1P lyase inhibition by 4‐deoxypyrodoxin (DOP) to raise S1P levels in vivo in an unicortical long bone defect model of posttraumatic osteomyelitis in mice. In a translational approach, human bone specimens of clinical osteomyelitis patients were treated in organ culture in vitro with DOP. Bone regeneration was assessed by μCT, histomorphometry, immunohistology and gene expression analysis. The role of S1P receptors was addressed using S1PR3 deficient mice. Here, we present data that DOP treatment markedly enhanced osteogenesis in posttraumatic osteomyelitis. This was accompanied by greatly improved osteoblastogenesis and enhanced angiogenesis in the callus accompanied by osteoclast‐mediated bone remodelling. We also identified the target of increased S1P to be the S1PR3 as S1PR3^−/−^ mice showed no improvement of bone regeneration by DOP. In the human bone explants, bone mass significantly increased along with enhanced osteoblastogenesis and angiogenesis. Our data suggest that enhancement of S1P/S1PR3 signalling may be a promising therapeutic target for bone regeneration in posttraumatic osteomyelitis.

## 1 INTRODUCTION

Posttraumatic osteomyelitis is a severe complication which occurs especially after open fractures.^1^ These bone infections often lead to large osseous defects or can even result in the amputation of the affected limb.^2^ Systemic diseases such as diabetes mellitus or peripheral vascular disease, increased age and immune deficiency markedly enhance the risk for bone infections.^3^, ^4^ Diagnosis of osteomyelitis can be difficult as clinical symptoms are often highly variable. Cardinal signs of infection such as pain, swelling and redness, and the presence of laboratory signs of inflammation hint towards an emerging bone infection.^5^ Although mandatory for bone infections, imaging procedures have a high false negative rate of up to 80% in early cases of osteomyelitis, whereas in microbiological analyses this rate amounts to 40%.^6^ Proper surgical debridement and antibiotic therapy are mandatory to treat osteomyelitis effectively.^7^ Importantly, the major importance of a thorough surgical debridement has been proven in multiple studies.^8^, ^9^, ^10^

Healing of an injured bone is regulated by highly complex inflammatory processes that when disturbed, lead to delayed bone healing or non‐unions. A decreased bone healing capacity after bone infections is common in a clinical context but the underlying mechanisms are largely unknown. We have established a murine osteomyelitis model of the murine long bone,^11^ where we observed decreased osteogenesis accompanied by a prolonged abacterial inflammatory reaction.^12^ Chemokines such as CCL2, CCL3 and CXCL2 and elevated B‐cell activity were involved in this process. We also showed that local application of adipose derived stromal cells (ASCs) and a recombinant Wnt3a agonist, respectively, were capable to overcome this impaired bone regeneration in vivo^13^, ^14^ and in vitro.^15^ Both ASCs and Wnt3a increased osteoblastogenesis and angiogenesis while decreasing osteoclast activity. While these treatment modalities were quite effective, a systemic approach to increase impaired bone regeneration is missing to date.

Sphingosine‐1‐phosphate (S1P) is a bioactive lipid with manifold effects on osteoblasts and osteoclasts as well as immune cells.^16^, ^17^ We have shown that pharmacological or genetic elevation of S1P in vivo exhibited a clear osteoanabolic effect in healthy mice and several osteopenia models.^18^ In this context a strong correlation between S1P activity and estrogen signaling became evident not only in bone tissue.^19^, ^20^ However, the impact of S1P on post‐infectious bone regeneration is unknown. Here, we investigated the effects of systemic pharmacological S1P elevation on bone regeneration in the murine posttraumatic osteomyelitis model in vivo and on cultured human bone specimens from osteomyelitis patients in vitro.

## 2 METHODS

### 2.1 Mouse posttraumatic osteomyelitis model

All experiments and procedures were in accordance to the Landesamt für Natur, Umwelt und Verbraucherschutz NRW, Germany (Permit Number: AZ 81‐02.04.2020.A075).

Twelve‐week‐old male and female C57BL/6J mice (Charles River Laboratories) as well as S1PR3^−/−^ (S1pr3t^m1Rlp^) and S1PR3^+/+^ mice (both on C57BL/6J background)^21^ underwent the following procedure^10^: Animals were housed in a gang with free access to water and food. After deeply anaesthetizing the animals, a skin incision was performed over the medial tibia exposing the proximal tibia plateau. Thereafter, a 1 mm hole was drilled into the tibia with a handheld drilling device (Ultimate 450, Nakanishi). *Staphylococcus aureus* was injected (1000 CFUs in 1 μL) into the bone defect, and the hole was sealed with bone wax prior to closing the wound. Two weeks after this surgical procedure, a second operation was performed. After skin incision, infected bone was exposed and necrotic, infected bone was debrided and thoroughly rinsed with sterile saline after which the wound was closed. 4‐deoxypyridoxine (DOP) was administered immediately thereafter via the drinking water at 180 mg/L. Control animals did not receive DOP. Mice were sacrificed 2 weeks after the second surgery and tibiae were collected for μCT, histological analysis and gene expression studies.

### 2.2 Human bone specimens

Bone specimens were collected from patients from a single institution. All patients selected suffered from an acute posttraumatic osteomyelitis of the tibial bone that was treated via segmental resection and bone transport. Absence of acute infection and bacterial colonization of the bone was verified by multiple microbiological analyses. Bone specimens from long bones were directly cultured in αMEM supplemented with 10% fetal bovine serum (FBS; Biochrom), 1% penicillin/ streptomycin (P/S; Invitrogen) at 37°C and 5% CO_2_ in the absence of presence of 200 μM DOP. μCT scans were performed at Day 0 and after 4 weeks of culture. Afterwards, bone specimens were fixed in 4% paraformaldehyde and further processed for histological analyses.

#### 2.2.1 Analysis of serum S1P levels

Whole blood of DOP treated and untreated animals with EDTA as anticoagulant was used to collect plasma. The blood was centrifuged at 1500 × g for 10 min at 4°C and the plasma was collected. Blood plasma S1P levels were determined by positive ionization using the LCMS‐8050 triple quadruple mass spectrometer (Shimadzu) as described previously.^22^

### 2.3 μCT analysis

Quantitative analysis of the defect area in mouse tibia and analysis of bone volume of human specimen was performed using a SkyScan X‐ray Microtomograph 1072 (SkyScan). For image acquisition, samples were placed in a tight plastic tube and acquisition was performed at 70 kV and 114 μA using 180° circular acquisition with steps of 0.45° between projections. For analysis of defect volume, tibiae were acquired using a pixel size of 11.32 μm, for human bone analysis acquisition of 18.88 μm pixel size was used. Images were reconstructed using the NRecon software (version 1.6.9.4; SkyScan), adequate corrections were applied. For calculation of bone and defect volume in mouse tibiae, 400 consecutive CT slides enclosing the defect area were analysed using the CTAnalyzer (version 1.18.9.0+; SkyScan). For analysis of the healing callus, a threshold of 50 is used, lamellar bone is analysed using a threshold of 60. Human bone samples were acquired before and after 28 days of cultivation. Total bone volumes were calculated using a threshold of 37. Analysis was performed according to the guidelines for assessment of bone microstructure in rodents using micro‐computed tomography.^23^

### 2.4 Histology, immunohistochemistry and immunofluorescence

Tibial murine bones and human bone specimens were fixed in 4% paraformaldehyde solution overnight, decalcified in 19% EDTA and embedded in paraffin. The bone was sectioned longitudinally at 9 μm with a microtome. Aniline blue, Trichrome and TRAP staining (TRAP Kit, Sigma‐Aldrich) were performed according to the manufacturer's instructions. Immunohistochemical staining was performed using the Vectastain ABC Kit (rabbit IgG, Vector Laboratories) using primary antibodies against Osteocalcin (FL‐95, cat. no. sc‐30045, Santa Cruz Biotechnologies). Antigen unmasking was performed using Proteinase K solution (Roche Diagnostics). Incubation in 3% hydrogen peroxide solution blocked endogenous peroxidase activity, while unspecific binding of primary antibody was blocked with normal serum. Primary antibody was applied at 4°C overnight. Primary antibodies against Runx2 (M‐70, cat. no. sc‐10758, Santa Cruz Biotechnologies) and CD‐31 (cat. no. 550274, BD Biosciences) were used for immunofluorescent staining. Initial steps were performed with the difference of the detection by secondary antibody. Here we used goat anti‐rabbit IgG conjugated to Alexa Flour 594 (Thermo Fisher Scientific). Zeiss Axivert 100 (Zeiss) was used for image acquisition. Quantification was performed via semi‐automated pixel quantification using Adobe Photoshop as previously described.^24^

### 2.5 qRT‐PCR

Tissue of tibial bone defects was homogenized and RNeasy mini kit (Qiagen) used for RNA isolation. TaqMan probes in StepOnePlus (Applied Biosystems) were used for quantitative polymerase chain reaction. Data of gene expression were normalized to housekeeping genes (*Gapdh* and 18S) and evaluated after ∆Ct method. Assessed genes were *Rankl*, *Opg*, *Tgf*‐*beta*, *Nfatc1*, *CD*‐*31*, *IL6*, *Mmp9*, *Osteopontin*, *Runx2*, *Wnt5a*, *Tnf*‐*alpha*, *Noggin*, *IL1RN*, *Fabp4*, *Glut4*, *Osteocalcin*, *Alp*, *IL1a and Vegf*‐*A*. Primer sequences were obtained with Primer‐Blast (NIH).

### 2.6 Study approval

All animal experiments and procedures were in accordance with the Landesamt für Natur, Umwelt und Verbraucherschutz NRW, Germany (Permit Number: AZ 81‐02.04.2020.A075). All procedures performed concerning human bone samples were in adherence to the local ethical committee. Written and informed consent was received prior to participation.

### 2.7 Statistical analysis

Statistical significance was set at a *p*‐value <0.05. A nonpaired two‐tailed *t*‐test was used for statistical analysis of differences between groups. Normality and equal variance was tested before analysis. Results were presented as mean ± standard error of the mean (SEM). Statistical significance was analysed using GraphPad Prism (version 9.3.1; GraphPad).

## 3 RESULTS

### 3.1 Pharmacological inhibition of the S1P lyase restores bone regeneration in posttraumatic osteomyelitis

**FIGURE 1:**
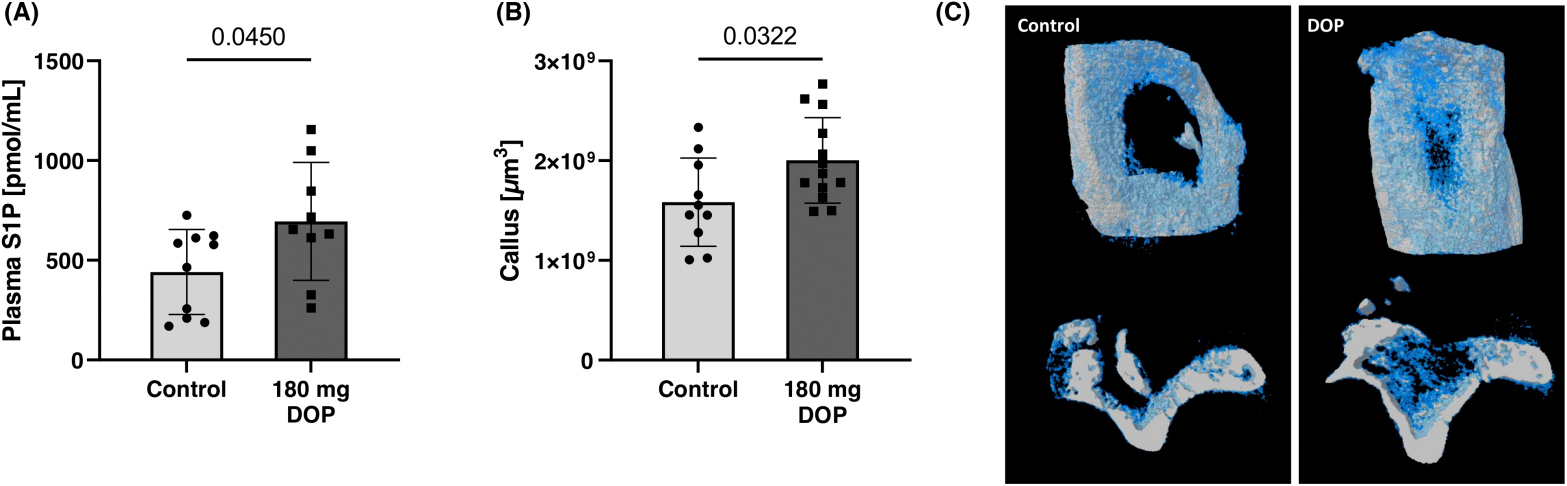
DOP treatment leads to increased S1P Plasma Levels and formation of callus during bone healing in C57Bl/6‐J mice 2 weeks after a uni‐cortical defect was created. (A) S1P levels in plasma after treatment with 180 mg/L DOP and control animals 2 weeks after a unicortical defect was created measured with mass spectrometry (*n* = 10 control, *n* = 9 DOP), and (B) Callus formation (A) and representative images (whitebone, Blue‐Callus) these mice mice analysed using micro‐computer tomography (*n* = 10 control, *n* = 13 DOP). Data are presented as mean ± SD and tested with two‐tailed *t*‐ test.

**FIGURE 2:**
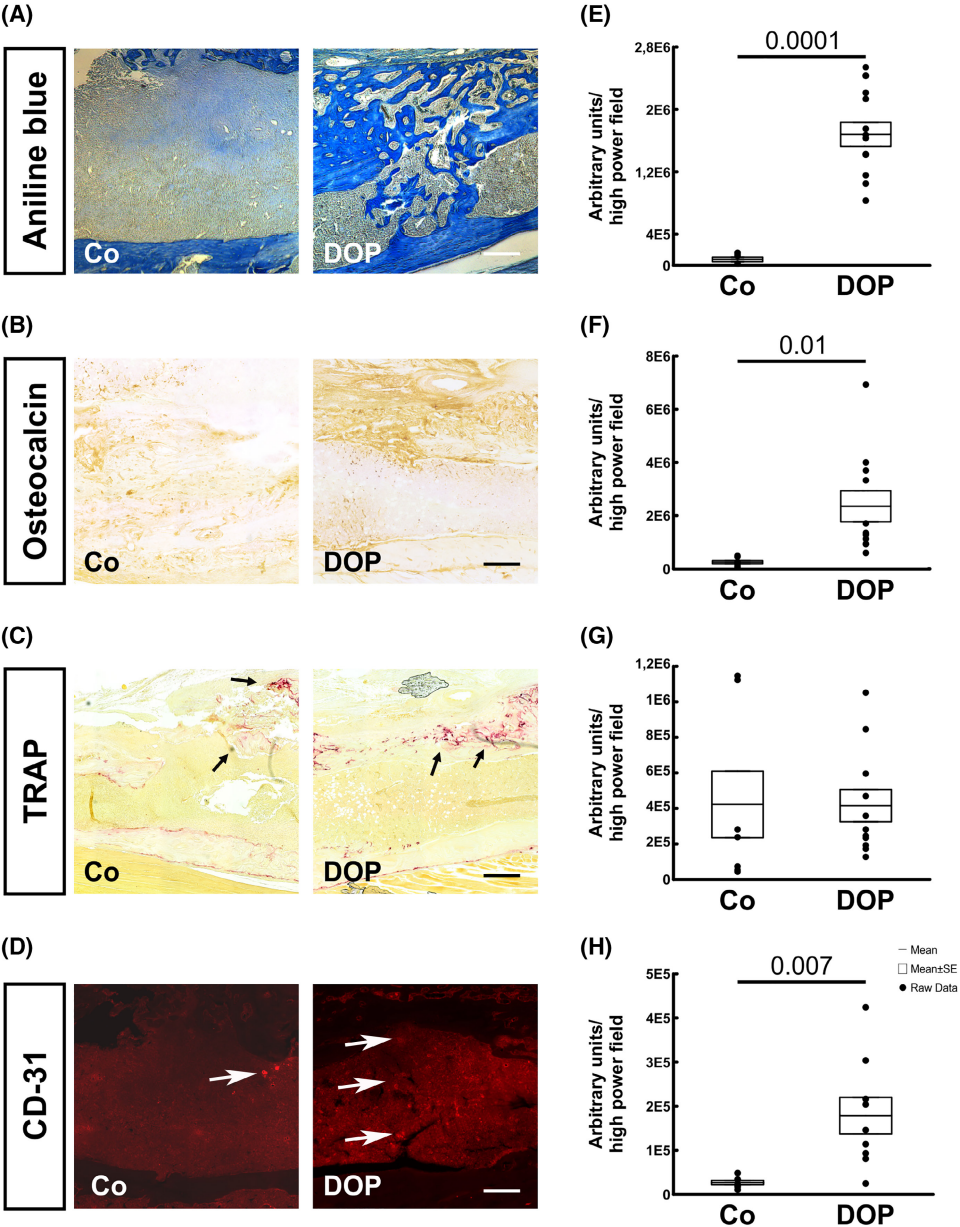
Representative aniline blue staining (A) and immunohistochemistry for (B) Osteocalcin, (C) TRAP and (D) CD31 with histomorphometrical quantification (E–H) in murine tibial defects 2 weeks postoperatively without (Co; *n* = 10) and with DOP treatment (DOP; *n* = 13). In all pictures the bony defect is shown with intact cortical bone at the bottom and monocortical defect at the top of each image. Black arrows in TRAP staining indicate stained osteoclasts and white arrows in CD‐31 staining indicate bloodvessels in the defect area. Results are shown as mean ± SEM. Scale bar: 200 μm.

To determine the effects of elevated S1P concentrations on osteogenesis after bone infection, S1P lyase was inhibited pharmacologically by oral 4‐deoxypyrodoxin (DOP) administration for 2 weeks starting immediately after debridement in our established murine posttraumatic osteomyelitis model.^11^ Serum S1P‐levels could be significantly increased via DOP treatment (Figure 1A). MicroCT scans of the bone defect area after 2 weeks showed significantly increased callus formation in the DOP group (1.3‐fold) in comparison to untreated controls (Figure 1B,C). Histomorphometrical analyses of the identical bones showed a dramatically larger callus (~15‐fold) at the defect site in the DOP group compared to controls (Figure 2A,E).

To elucidate the underlying mechanisms, we assessed the effects on osteoblasts and osteoclasts in the bone defect area. We observed that the numbers of mature osteocalcin‐positive osteoblasts were substantially (9.5‐fold) higher within the defect area of DOP treated mice (Figure 2B,F). TRAP‐positive osteoclasts were similar in both groups. Furthermore, CD31 positive staining was also increased (6.7‐fold) in the DOP treated mice indicative of enhanced angiogenesis (Figure 2C–H).

### 3.2 Increased expression of osteogenic and angiogenic genes in DOP treated bone defects

**FIGURE 3:**
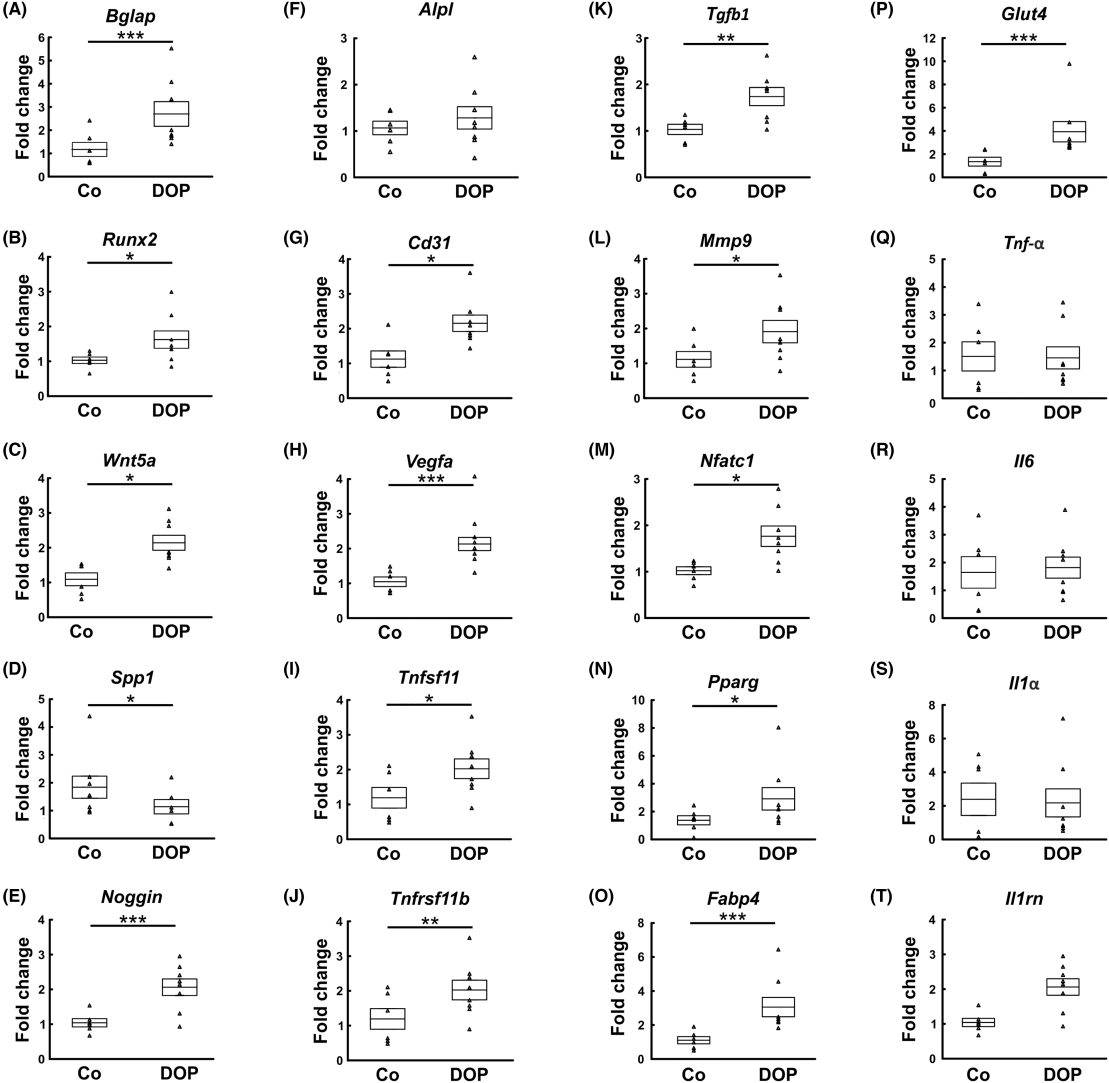
Quantitative RT‐PCR analysis of osteoblast related genes Bglap (A), Runx2 (B) and Wnt5a (C); osteogenic genes Spp1 (D), Noggin (E) and Alpl (F); angiogenesis related genes Cd31 (G) and Vegfa (H); osteoclast related genes Tnfsf11 (I), Tnfrsf11b (J) Tgfb1 (K), Mmp9 (L), Nfatc1 (M); adipogenic differentiation genes Pparγ (N), Fabp4 (O) and Glut4 (P), and inflammatory markers Tnf‐alpha (Q), Il6 (R), Il1a (S), and Il1rn (T) (n = 7 control and DOP each). Data are presented as mean ± SEM. *p*‐value: * < 0.05, ** < 0.01, *** < 0.001.

We observed that expression of *Bglap* (Osteocalcin), *Runx2* and *Wnt5a* as important markers for osteoblast differentiation and function were significantly increased in bone defect tissue from the DOP group compared to controls (Figure 3A–C). Furthermore, osteogenic markers such as *Spp1* (Osteopontin) and *Noggin* were also increased, whereas *Alpl* was unaltered (Figure 3D–F). In agreement with the higher CD31 positivity on immunostaining, *Vegfa* and *Cd31* expression was increased in DOP treated bone defects (Figure 3G,H). In addition, Tnfsf11 (*RANKL*) and Tnfrsf11b (*OPG*) expression along with that of *Tgfb1*, *Mmp9* and *Nfatc1* was also significantly upregulated in DOP treated tissue (Figure 3I–M). In contrast, expression of inflammatory markers such as *Tnf*‐*a*, *Il6*, *Il1a*, and *Il1rn* did not differ (Figure 3Q–T). Interestingly, important markers for adipogenic lineage differentiation such as *Pparγ*, *Fabp4* and *Glut4* were significantly upregulated at the transcriptional level after DOP treatment (Figure 3N–P).

### 3.3 S1PR3 deficiency abolishes enhanced bone regeneration by DOP treatment

**FIGURE 4:**
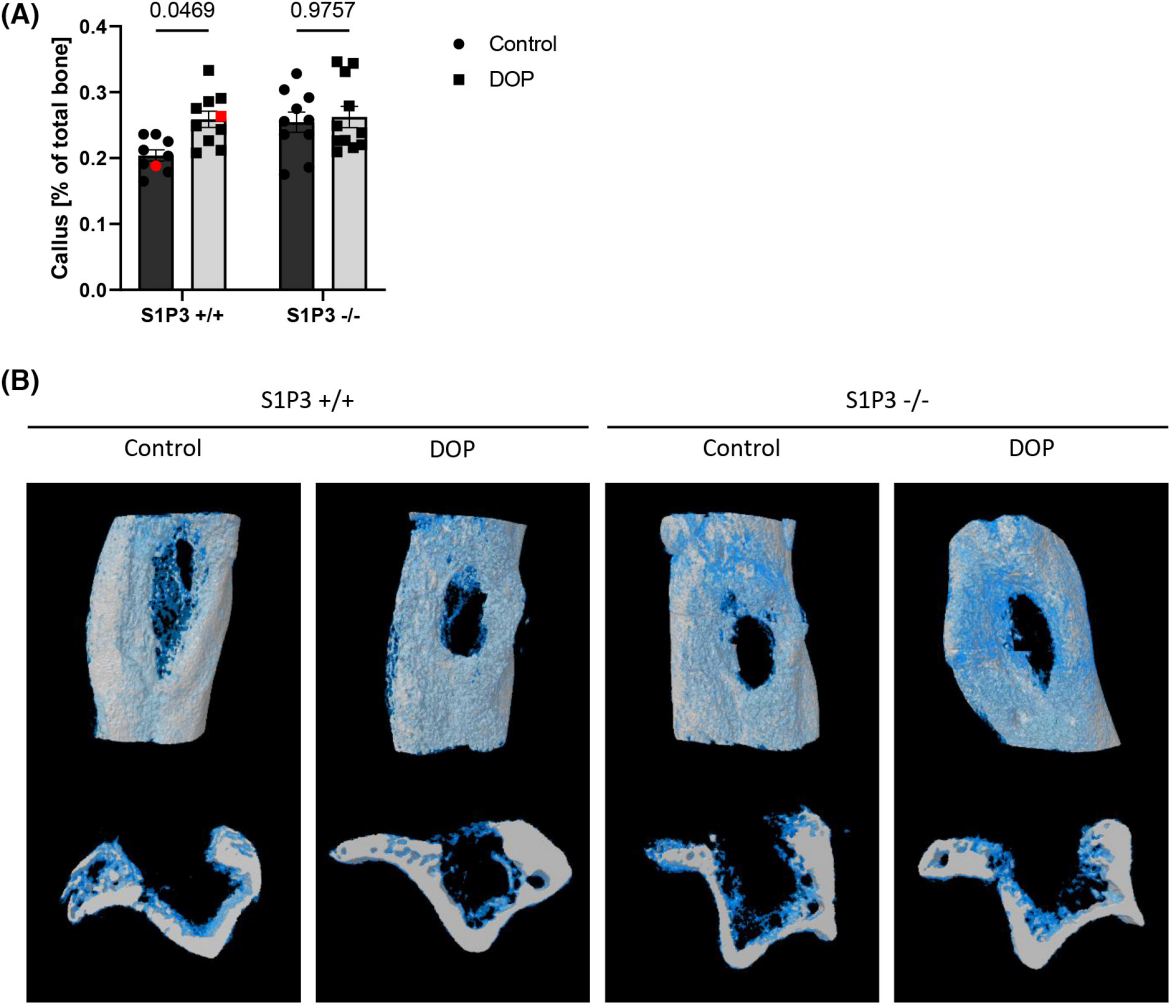
DOP treatment leads to increased formation of callus during bone healing in S1P3+/+ mice 2 weeks after uni‐cortical bone defect was created. This effect is prevented in S1P3−/− receptor KO mice. (A) Callus formation (*n* = 9/10/10/11) and (B) representative images (white‐ bone, blue‐callus) of WT and KO mice treated with 180 mg/L DOP for 2 weeks and according controls were analysed using micro‐ computer tomography. Data are presented as mean ± SEM and tested with two‐way anova.

**FIGURE 5:**
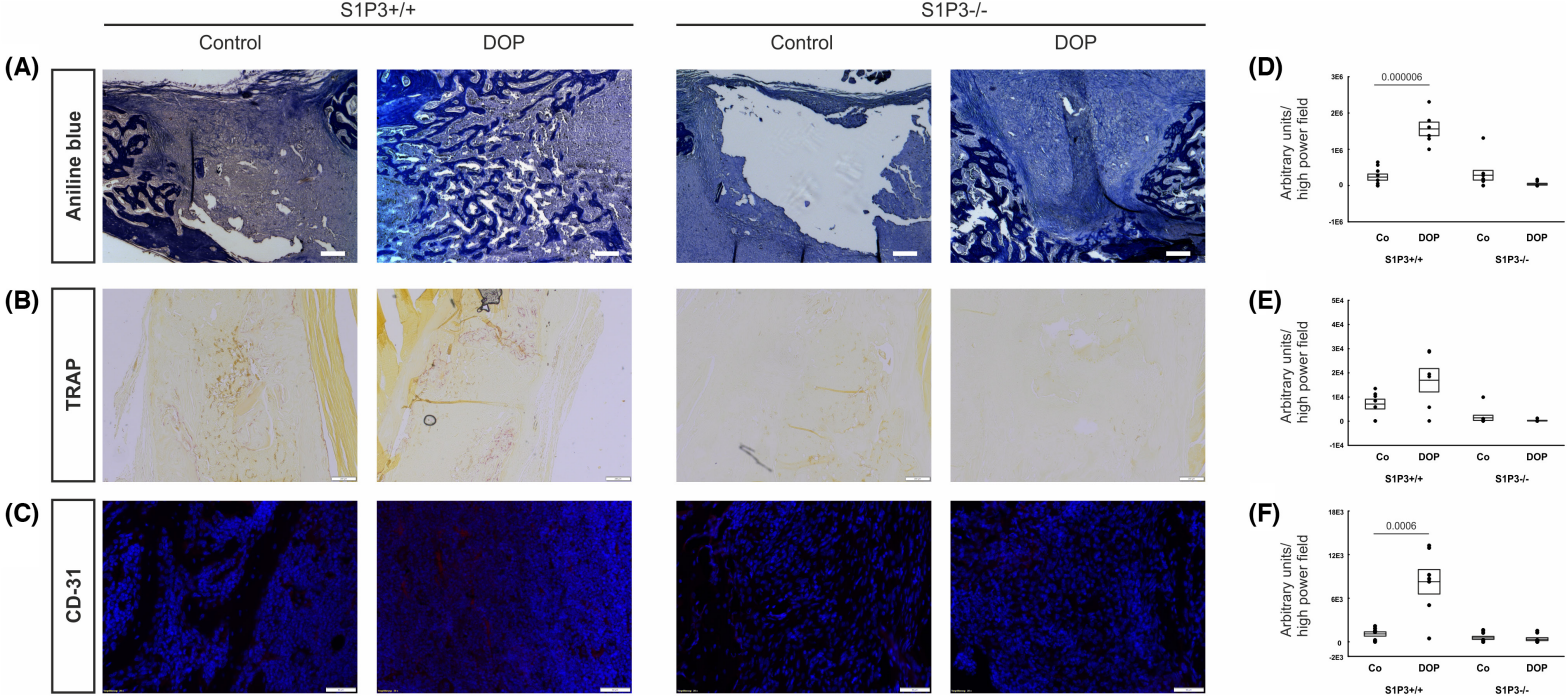
Stainings for aniline blue staining (A) and immunohistochemistry for (B) TRAP and (C) CD31 and according histomorphometrical quantification (D–F) of S1P3 receptor deficient knockout mice (S1P3−/−) (*n* = 11) and wildtype control (S1P3+/+) (*n* = 10) 2 weeks postoperatively without (Co) and with DOP treatment (DOP). Results are shown as mean ± SEM. Scale bar: 200 μm in aniline blue and TRAP staining and 100 μm in CD‐31 staining.

Having identified a beneficial effect of DOP treatment on osteomyelitic bone regeneration, we performed experiments to unravel the involvement of S1P receptors. As S1P/S1PR3 signalling has been implicated in enhanced osteogenesis^25^ we employed S1PR3^−/−^ and S1PR3^+/+^ mice in the same model. Strikingly, S1PR3^−/−^ were completely resistant to the bone regenerative effect of DOP whereas the familiar increase in bone formation was present in S1PR3^+/+^ controls as determined by μCT analysis (Figure 4) and histomorphometry on aniline blue stained bone defects (Figure 5A,D). Neither was there an increase in CD31 immunostaining after DOP treatment in S1PR3^−/−^ mice (Figure 5C,F). Histological evaluation of osteoclasts by TRAP staining showed a markedly reduced activity in both untreated and DOP treated S1PR3^−/−^ mice (Figure 5B,E).

### 3.4 Regenerative capacity of human osteomyelitic bone is improved by DOP treatment in vitro

**FIGURE 6:**
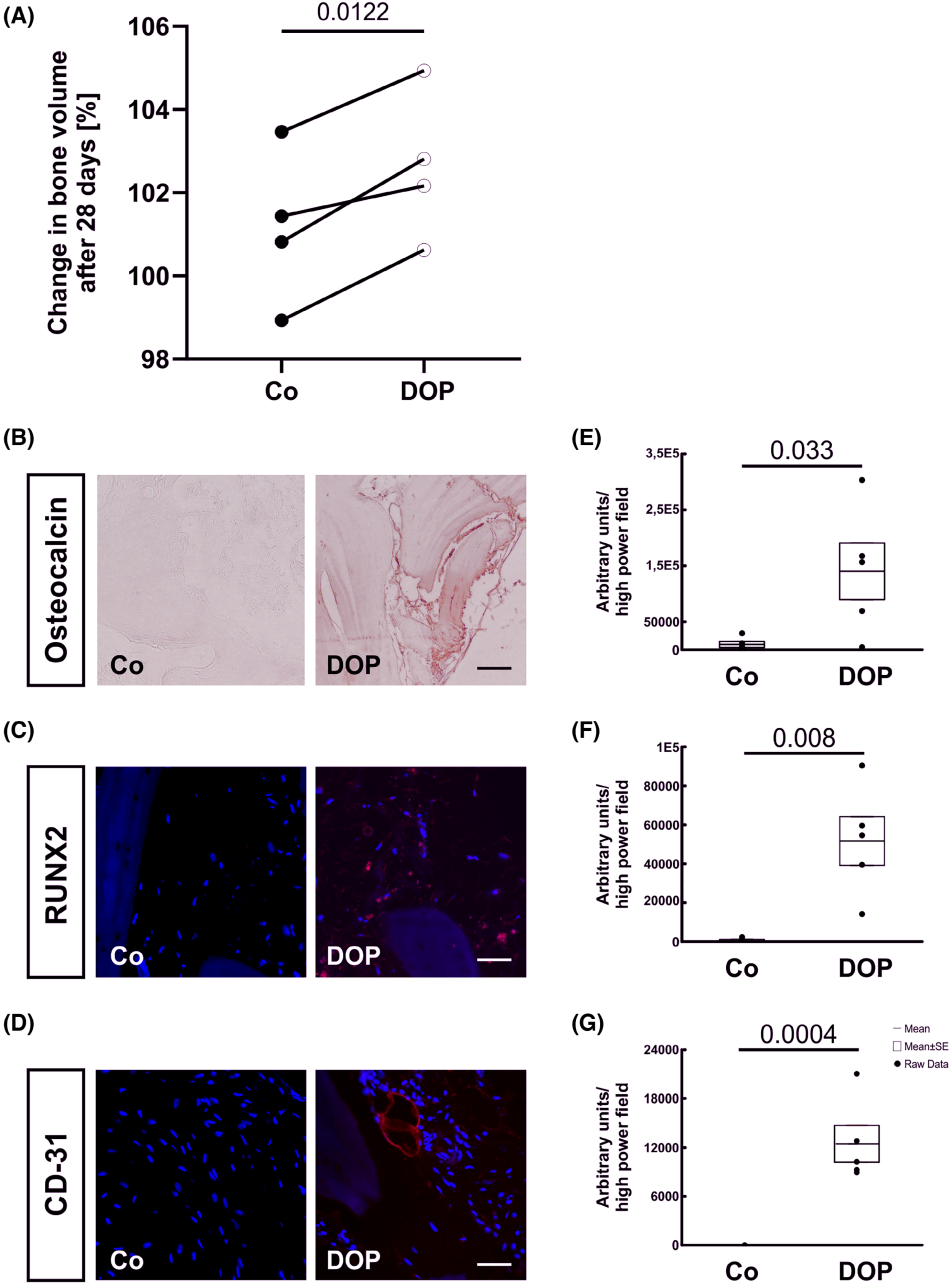
(A) Percentage of changes in bone volume of control and 0.2 mM DOP treated human osteomyelitic bone samples after 4 weeks of tissue culture analysed using micro‐computer tomography (*n* = 5) and immunohistochemistry for (B) Osteocalcin, (C) Runx2 and (D) CD‐31 (E‐G) (*n* = 7). Results are shown as mean ± SEM. ns, not significant, *p*‐value: * < 0.05, *** < 0.001. Scale bar: 200 μm in Osteocalcin staining and 100 μm in Runx2 and CD31 staining.

In a translational approach, we tested whether the pro‐regenerative DOP effect may also apply in human bone after osteomyelitis. To do this, we explanted specimens of human long bones after infection and cultured corresponding samples in organ culture without and with DOP supplementation for 4 weeks. Bone specimens were scanned by μCT analysis before and at the end of organ culture and processed for histology. Indeed, we observed significantly increased bone volume in bones cultured in the presence of DOP as compared to specimens from the same individual but cultured without DOP (Figure 6A). Immunohistomorphology for osteocalcin, and Runx2 also showed 14.8‐ and 73.5‐fold increases in the DOP treated group compared to controls (Figure 6B,C,E,F). Interestingly, DOP treated samples showed a markedly enhanced angiogenesis in CD31 immunoflourescence, while untreated samples showed no signalling (Figure 6D,G).

## 4 DISCUSSION

Bone infections are a major burden for affected patients and a challenging clinical issue as the goals are not only to effectively treat the infection but also to restore the impaired bone healing capacity. The present work aimed to evaluate the effect of S1P on bone regeneration after posttraumatic osteomyelitis. Indeed, we found that inhibition of S1P degradation by the S1P lyase enhanced osteogenesis in postinfectious murine long bones in vivo. We could also translate this to postinfectious human bones in organ culture. We have previously described that Increasing S1P concentrations in vivo has an osteoanabolic effect in preclinical models of osteopenia and osteoporosis through stimulation of osteoblastogenesis and suppression of osteoclastogenesis.^18^ The osteogenic effect occurs mainly through the S1P receptor 2 (S1PR2), which favours the osteoblastic lineage of progenitor cells over adipogenic differentiation.^18^, ^26^, ^27^ Here, we have also observed increased osteogenesis but adipogenic markers such as *Fabp4*, *Glut4*, *Pparγ* were also increased. However, Pparγ not only affects adipogenesis but is an important mediator of osteoclastogenesis through *c*‐*fos*.^28^, ^29^ Accordingly, *Pparγ*‐knockout mice exhibit severe osteopetrosis underlining the importance of osteoclasts for physiological bone turnover.^28^ In our study, osteoclast activity was higher as evidenced by increased expression of RANKL and MMP9, two important factors for osteoclastogenesis.^30^, ^31^ Thus, we believe that the induction of Pparγ in our study mirrors the remodelling phase of newly formed bone where resorptive processes are highly important. Accordingly, we suggest that in regenerative osteogenesis, S1P acts in an osteoanabolic manner and promotes physiological bone resorption during the remodelling.

Our second important observation was the increase in angiogenesis in the callus by S1P lyase inhibition. Whereas S1P is well known pro‐angiogenic factor in general,^32^, ^33^ there is also evidence of its important role in bone angiogenesis that then drives osteogenesis. Recently, a new capillary subtype has been identified in bone that connects angiogenesis and osteogenesis and is reduced in osteopenic aged mice.^34^ These so‐called H type vessels associate with osteoprogenitors and restore bone mass in osteopenic mice when stimulated. A current study, which showed improved fracture healing after controlled mechanical loading, found evidence for enhanced bone formation through H‐Type vessels in a S1PR1‐dependent manner.^35^ In contrast, much less is known of S1P in the context of angiogenesis in bone regeneration after injury: only a single study on bone tissue engineering has shown a pro‐angiogenic effect of S1P in a calvarial defect model.^36^, ^37^ We believe this issue to be highly relevant as angiogenesis is crucial for structural bone restoration after infection. Osteoblastogenesis has been shown to be regulated by S1PR2^18^, ^26^, ^27^ and S1PR3.^25^ In our present study, we have observed that the benefit of S1P lyase inhibition on bone regeneration in posttraumatic osteomyelitis was abolished in S1PR3 deficient mice. This is supported by studies showing that the S1P analogon fingolimod increased bone thickness in wild type but not S1PR3 deficient mice.^25^ Moreover, antagonizing S1PR3 in MC3T3 cells resulted in decreased AP activity and *Runx2* expression.^38^ Interestingly, we also observed reduced osteoclasts in the regenerating bone of S1PR3 deficient mice with and without S1P lyase inhibition. This suggests that S1PR3 is involved in the physiological osteoclast‐dependent remodelling processes. S1PR3 is expressed on the surface of mature osteoclasts^39^ but its specific functions are less well characterized in contrast to osteoclast S1PR1 and S1PR2.^39^, ^40^ Whether reduced osteoclast function in our study there is a direct effect through osteoclast S1PR3 or as a consequence of reduced bone formation due to impaired osteoblastogenesis remains to be addressed. As already mentioned, S1P‐Signalling strongly correlates with oestrogen activity in bone formation.^19^ Oestrogen has been shown to activate EGFR via S1PR3^20^ which in turn promotes bone formation.^41^ Considering that estrogen signaling is well established in bone tissues, a possible link between enhanced S1P signalling, promoted by DOP therapy, and oestrogen could be hypothesized. Of course, this needs to be further validated in future studies. Furthermore, the role S1PR1, which is known to promote estrogens' effects, should be emphasized in previous studies.^42^

From a clinical point of view, S1P signalling has caught attention in inflammatory bone loss and resorption: Synovial fluid of patients with rheumatoid arthritis (RA) and extensive bone loss have significantly higher levels of S1P compared to patients with osteoarthritis,^43^ and S1P levels in serum correlated with inflammatory activity and bone erosion in patients with periodontitis.^44^ The S1P analogon Fingolimod (Gilenya®) is clinical used for relapsing multiple sclerosis^45^, ^46^ and currently under investigation for several autoimmune diseases.^47^ In a combined model of osteoporosis and rheumatoid arthritis mimicking female RA patients, fingolimod increased bone mineral density and reduced inflammatory activity more efficiently than corticosteroids.^48^ Fingolimod has also reduced inflammatory activity in a collagen induced arthritis model in rodents.^49^, ^50^ This would be appealing to test Fingolimod and other pharmacological S1P receptor agonists on post‐infectious bone regeneration in the future.

## 5 CONCLUSIONS

In summary, our study has demonstrated clear acceleration of bone regeneration in a murine model of posttraumatic osteomyelitis by pharmacological S1P lyase inhibition and has validated these observations in post‐infectious human bone. We have identified S1PR3 to be instrumental in mediating these effects that were based on increased osteogenesis, enhanced bone remodelling and stimulation of angiogenesis in the callus. We believe that our data may provide new translational implications for the therapy of impaired bone regeneration after traumatic defects.

## AUTHOR CONTRIBUTIONS

**Johannes Maximilian Wagner:** Conceptualization (equal); funding acquisition (equal); project administration (equal); writing – original draft (equal); writing – review and editing (equal). **Annalena Wille:** Data curation (equal); formal analysis (equal); methodology (equal); writing – review and editing (equal). **Maria Fueth:** Data curation (equal); formal analysis (equal); investigation (equal). **Sarah Weske:** Formal analysis (equal); investigation (equal). **Sebastian Lotzien:** Conceptualization (equal); data curation (equal); resources (equal). **Felix Reinkemeier:** Conceptualization (equal); project administration (equal); software (equal); supervision (equal). **Christoph Wallner:** Validation (equal); visualization (equal); writing – review and editing (equal). **Alexander Sogorski:** Investigation (equal); methodology (equal). **Stephanie Dittfeld:** Data curation (equal); formal analysis (equal); resources (equal). **Mustafa Becerikli:** Project administration (equal); supervision (equal). **Thomas Schildhauer:** Project administration (equal); supervision (equal). **Marcus Lehnhardt:** Conceptualization (equal); funding acquisition (equal); project administration (equal); resources (equal). **Bodo Levkau:** Project administration (equal); supervision (equal); validation (equal); writing – original draft (equal); writing – review and editing (equal). **Björn Behr:** Conceptualization (equal); data curation (equal); funding acquisition (equal); project administration (equal).

## CONFLICT OF INTEREST STATEMENT

The authors declare they have no conflict of interest.

## Data Availability

not applicable
